# Involvement of *ACSM* family genes in the reprogramming of lipid metabolism within tumors and their investigation as promising therapeutic targets

**DOI:** 10.3389/fcell.2025.1663033

**Published:** 2025-09-26

**Authors:** Ke-Xin Fang, Zi-Qi Li, Jin-Liang Dong, Ze Yu

**Affiliations:** ^1^ The Laboratory of Cytobiology and Molecular Biology, Zhoushan Hospital, Wenzhou Medical University, Zhoushan, Zhejiang, China; ^2^ Department of General Surgery, Zhoushan Hospital, Wenzhou Medical University, Zhoushan, Zhejiang, China

**Keywords:** ACSM family, lipid metabolic reprogramming, fatty acid activation, tumors, therapeutic targets

## Abstract

Metabolic reprogramming of tumor cells is an important feature of oncogenesis and progression, with lipid metabolism playing a key role in this process. The enzymes encoded by the ACSM (Acyl-CoA Synthetase Medium-Chain Family) genes play a crucial role in fatty acid activation, affecting the energy supply and membrane synthesis of tumor cells. In recent years, research on the expression patterns of ACSM family genes in different tumor types and their regulatory mechanisms has gradually increased, revealing their close relationship with tumor development. However, current research on ACSM family genes still has some shortcomings, such as a lack of systematic large-scale clinical data support and an in-depth understanding of their regulatory networks. This work summarizes the role of ACSMs in tumor lipid metabolic reprogramming and explores their research progress as potential therapeutic targets, providing new ideas for future tumor treatment.

## 1 Introduction

Metabolic reprogramming of tumor cells is one of the important characteristics of malignancies, especially during the occurrence, development, and metastasis of tumors, where lipid metabolism reprogramming plays a key role (Yang et al., 2023; Wang et al., 2024; Liu et al., 2023; Liu et al., 2025). Recent years, an increasing number of studies have shown that ACSM (Acyl-CoA Synthetase Medium-Chain Family) gene family is significant in regulating tumor cell metabolism. These enzymes encoded by the ACSMs are responsible for activating medium-chain fatty acids, converting them into acyl-CoA, and participating in key steps such as β-oxidation of fatty acids, triglyceride synthesis, and membrane phospholipid synthesis (Boomgaarden et al., 2009; van der Sluis and Erasmus, 2016). This metabolic reprogramming enables tumor to adapt nutrient-deficient microenvironment, meeting their rapid proliferation needs.

The function of ACSM enzyme under normal physiological conditions is mainly reflected in the activation process of medium-chain fatty acids, and its molecular mechanism involves the acylation reaction of fatty acids, which is an important prerequisite for fatty acids to enter the synthesis and degradation metabolic pathways (van der Sluis and Erasmus, 2016). Specifically, ACSM promotes the activation of fatty acids by combining them with coenzyme A, enabling them to participate in biochemical reactions of energy metabolism and lipid synthesis. These enzymes not only directly participate in the metabolism of fatty acids but also interact synergistically with other lipid metabolic enzymes, forming a complex regulatory network. For example, the interaction of ACSM enzyme with fatty acid synthase (FAS) and other related enzymes in cells promotes the balance of lipid synthesis and degradation, thereby maintaining the energy homeostasis and physiological balance of cells.

The ACSM gene family mainly includes seven members: ACSM1, ACSM2A, ACSM2B, ACSM3, ACSM4, ACSM5, and ACSM6 (Supplementary Table S1). The enzymes encoded by these genes play an important role in fatty acids metabolism (Watkins et al., 2007). There are significant functional differences among the subtypes based on different tissue distributions and substrate specificities. For example, ACSM1 is primarily expressed in the liver, while ACSM2A is dominant in muscle tissue. The different distributions of these subtypes allow them to exert their unique physiological functions in the body’s lipid metabolism. In addition, the analysis of the conservation of gene structure and protein functional domains of the ACSM gene family indicates that these genes have retained important functional features during evolution, such as the AMP-binding domain (van der Sluis and Erasmus, 2016). This conservation not only reflects their key role in lipid metabolism but also provides the foundational support for understanding their functions under different physiological states. This study will focus on ACSMs, reviewing and discussing the mechanisms and targets of ACSMs in various tumors.

## 2 The functions and activation of fatty acids (FA)

**FIGURE 1:**
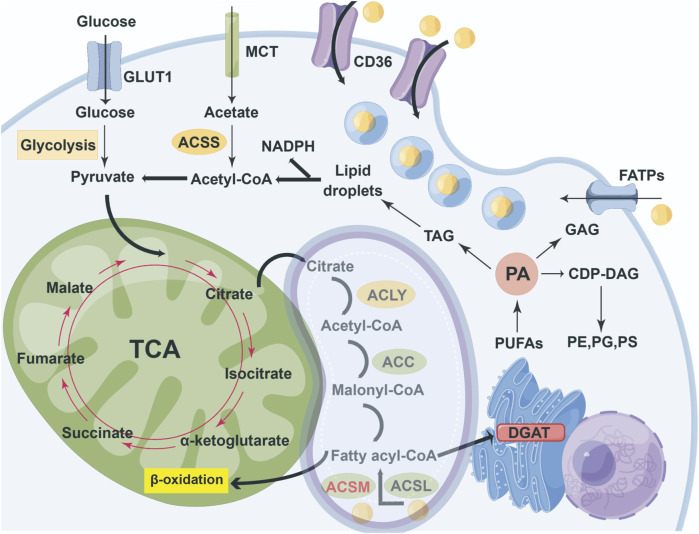
Overview of cellular partitioning of fatty acids. The key pathways of intracellular carbon source metabolism and lipid synthesis integrate the uptake and metabolism of glucose, acetic acid, and exogenous fatty acids, as well as the synthesis and storage processes of lipids, A fatty acid entering cells is rapidly 'activated' by acyl- CoA synthetases (ACSM and ACSL) to form fatty acyl-CoA. A 'pool' of fatty acyl-CoAs might either enter the mitochondrion for β-oxidation, or enter the endoplasmic reticulum for triacylglycerol synthesis by DGAT enzymes. Most of the ACSMs are located in mitochondria and, under certain circumstances, transfer to the cytoplasm to exert their functions.

Fatty acids fulfill a multitude of functions in living organisms. Primarily, they act as foundational substrates for the synthesis of structural lipids, including phospholipids and sphingolipids, which constitute essential components of cellular membranes. Additionally, when the oxidation of fatty acids is unnecessary for energy production, these compounds can be utilized as precursors for energy-storing lipids, such as triglycerides and cholesterol esters. Concurrently, fatty acids can undergo β-oxidation, thereby supplying energy to cells (Ma et al., 2018). Ultimately, fatty acid derivatives can be transformed into various signaling molecules, like diacylglycerol, which play crucial roles in mediating cellular processes (Ma et al., 2019). The distribution and metabolic pathways of fatty acids in cells are shown in Figure 1.

The initial phase of FA metabolism involves the activation of FA, which encompasses two distinct steps (Jencks and Lipmann, 1957; Mahler et al., 1953), the first step entails the adenylylation of the substrate, relying on adenosine triphosphate (ATP) and resulting in the release of pyrophosphate (PPi); subsequently, coenzyme A (CoA-SH) displaces the adenosine moiety and establishes a thioester bond with the substrate, with the adenosine component liberated as adenosine monophosphate (AMP).

**Step I:** fatty acid+ATP → fatty acyl−AMP+PPi

**Step II:** fatty acyl−AMP+CoA−SH → fatty acyl−S−CoA+AMP

**Total reaction equation:** fatty acid+ATP+CoA−SH →fatty acyl−S−CoA+AMP+PPi

## 3 The functions of *ACSM* family genes in different tumors

### 3.1 The functions and positioning of ACSMs

#### 3.1.1 ACSM1 (BUCS1, MACS1, HMX-B)

*ACSM1* gene is situated at the locus of chromosome 16p12.2 (Watkins et al., 2007). ACSM1 was initially extracted from human liver mitochondria (Fujino et al., 2001), However, subsequent studies have shown that the expression level of ACSM1 is the highest in the testicles, followed by the ovarian and pancreatic tissues, while it is relatively low in the initially discovered liver. Predominantly found within the mitochondrial matrix, ACSM1 possesses medium-chain fatty acid: CoA ligase activity and displays a wide substrate specificity for fatty acids ranging from 4 to 16 carbon atoms. Acyl-CoA produced is chiefly utilized for β-oxidation, facilitating the generation of ATP and CO_2_ within the mitochondrial matrix [ (Fujino et al., 2001; Fouché et al., 2025; Tan et al., 2025)]. Additionally, ACSM1 demonstrates activity towards carboxylic exogenous compounds like benzoates, earning it the alternate designation of xenobiotic: CoA ligase or XM ligase (Fujino et al., 2001). Among its substrates, ACSM1 shows greatest efficiency with hexanoate, followed by octanoate and benzoate, while its activity is comparatively lower with salicylate and laurate, thereby primarily reflecting its role as a medium-chain fatty acid activating enzyme. Besides, ACSM1 is capable of activating lipoic acid, which is crucial for the utilization of lipoic acid sourced from the salvage pathway (Paredes et al., 2018).

#### 3.1.2 ACSM2A (MACS2) and ACSM2B (HXM-A)

*ACSM2* gene comprises two distinct loci, namely, *ACSM2A* and *ACSM2B*. The coding sequences of the 2 genes exhibit a remarkable 98.8% similarity, while their amino acid sequences share an impressive 97.6% identity, both resulting in a protein chain of 577 amino acids (Watkins et al., 2007). Notably, *ACSM2A* & *ACSM2B* genes are genetically well-conserved (Van Der Sluis, 2018). Although the biological role of ACSM2A remains uncharacterized across any species, crystalline structure of ACSM2A has been deciphered as a complex with various substrates, products, and cofactors (Kochan et al., 2009). ACSM2B, initial isolated from human liver mitochondria, exhibits ability to activate exogenous carboxylic acids and medium-chain fatty acids, displaying the peak activity against benzoates, followed by medium-chain fatty acids, and showing lesser activity against salicylic, primarily functioning as an exogenous carboxylic acid-activating enzyme (Vessey et al., 2003; Vessey et al., 1999).

#### 3.1.3 ACSM3 (SAH/SA)

ACSM3 has medium-chain fatty acid: CoA ligase activity and is highly likely to be located in the mitochondria. Report suggests that Acsm3 knockout mouse exhibited abnormal mitochondrial morphology, decreased ATP contents and enhanced ROS levels in their livers (Junková et al., 2021; Xiao et al., 2024). Isobutyric acid is preferred fatty acid for ACSM3 protein among C2-C6 fatty acids, and the activated fatty acids are mainly used for oxidation to produce ATP and CO_2_ in the mitochondrial matrix.

The expression of *ACSM3* can be regulated by various factors. Butyrate can increase the expression of *ACSM3* in cultured adipocytes (Wang et al., 2020). *ACSM3* is downregulated in the kidneys of mice with chromium accumulation (Sun et al., 2021), and downregulated in the visceral adipose tissue of mice on a high-fat diet (Choi et al., 2015), while nitrogen oxide Tempol can upregulate *ACSM3* in the adipose tissue of high-fat diet mice (Choudhuri et al., 2022). Rutin significantly increased the expression of *ACSM3* mRNA in the brown adipose tissue and white adipose tissue of db/db mice, which is beneficial for inducing thermogenesis in adipocytes (Cheng et al., 2022). The expression of ACSM3 is continuously upregulated in the liver of early postpartum cows, suggesting that ACSM3 may be involved in the process of females experiencing negative the energy balance in early postpartum to meet lactation demands (Singh et al., 2019). Oar-miR-655-3p induced the downregulation of ACSM3 expression leads to abnormal muscle development in sheep (Sun et al., 2019). Melatonin treatment during cisplatin-induced acute kidney injury in mice increased the expression of *ACSM3*, a gene related to the fatty acid oxidation (FAO) pathway, by upregulating PPARα expression (Sun et al., 2022).

#### 3.1.4 ACSM4

*ACSM4* is situated on chromosome 12 and encodes a protein comprising 580 amino acids, which is expressed at minimal levels within testis (Watkins et al., 2007). Among the genes encoding mitochondrial proteins (NEMPs) in the nucleus, multiple SNPS in two NEMP genes are significantly associated with the progression of AIDS: The acyl-coA synthase midchain family member 4 (ACSM4) and the peroxisome D3, D2-ene-kyiv enzyme a isomerase (PECI), thus, in the context of diseases, the genetic polymorphisms of ACSM4 are significantly associated with the rapid progression of AIDS (HIV) and may mediate immune regulation by influencing mitochondrial function (Hendrickson et al., 2010).

#### 3.1.5 ACSM5 (MACS3)

The *ACSM5* gene is situated on human chromosome 16p12.3, coding for a protein composed of 579 amino acids, predominantly expressed in the kidneys and liver of humans (Iwai et al., 2003). To date, the enzymatic role of ACSM5 remains uncharacterized.

#### 3.1.6 ACSM6

*ACSM6*, distinct from its counterparts within ACSM family, is situated at position 10q23.33 on chromosome 10 and exhibits minimal expression predominantly within the pancreas (Watkins et al., 2007). This enzyme facilitates the attachment of fatty acids to coenzyme A in the process of β-oxidation, functioning upstream of carnitine acylation, and plays a crucial role in the transport of fatty acids into mitochondria (Langley et al., 2013). While ACSM6 has been identified in humans and is expressed in the brain, it is notably absent in rodent species, hinting at potential cognitive advantages in biological organisms (Fernandez and Ellis, 2020). To date, research on the functional dynamics of ACSM6 protein remains sparse, with no significant studies linking it to human diseases having been published.

### 3.2 The role of ACSMs in different tumors

#### 3.2.1 ACSMs in breast cancer

In the study of specific markers for the invasive apocrine carcinomas (IACs), it is pointed out that the combination of ACSM1 and 15-prostaglandin dehydrogenase defines IAC as a unique and heterogeneous molecular subtype of breast cancer (Celis et al., 2008; Celis et al., 2009). In mammals, ACSM1 can be degraded through the mitochondrial degradation pathway mediated by caseinolytic peptidase (Clp)-protease complex, a process regulated by Poldip2 (Paredes et al., 2018). Further studies found that, similar to hypoxic cells, a decrease in Poldip2 leading to ACSM1 degradation was also observed in triple-negative breast cancer (TNBC), and the degradation of ACSM1 impairs the acylation of prolyl-4-hydroxylase (PHD) and α-ketoglutaric dehydrogenase (αKDH) complex, limiting mitochondrial respiration, thereby inducing stable expression of hypoxia-inducible factor 1α (HIF-1α), resulting in adaptive metabolic reprogramming of cancer cells to hypoxia, demonstrating that the Poldip2/ACSM1 axis is crucial in the process of hypoxia and cancer cell metabolic adaptation (Paredes et al., 2018).

ACSM5 is lowly expressed in breast cancer tumor, and its expression is associated with poor prognosis in patients, making it a potential biomarker for prognosis and diagnosis. In addition, there is a positive correlation between ACSM5 and LINC00265, indicating that there may be a regulatory mechanism between them in breast cancer (Yazdani et al., 2021).

#### 3.2.2 ACSMs in liver cancer

The concentration of all ACSM in human livers is 3 to over 400 times that of liver cancer cell lines. Among them, early studies have shown that ACSM2B is the main transcript of the ACSM gene family in human livers (Boomgaarden et al., 2009). The downregulation of ACSM5 in liver cancer tissues and cell lines is mediated by DNA methylation. Research has found that the use of DNMT1 inhibitors can reduce the methylation level in the ACSM5 promoter region, thereby effectively increasing its expression (Yang et al., 2024). With the advancement and development of detection technology, further research has shown that ACSM2A is the main transcript in the human liver, followed by ACSM2B and ACSM5.

The expression of *ACSM3* is diminished in liver cancer tissues and progressively declines with the advancement of the disease. This reduced ACSM3 portends a grim prognosis for individuals afflicted with liver cancer (Ding et al., 2022; Gopal et al., 2017). Concurrently, ACSM3 levels in patients are linked to factors like high alpha-fetoprotein and alanine aminotransferase, younger age, more tumor nodules, and larger tumor volume. Its expression in HCC is regulated by HNF4α and inhibited by PPARγ, with TGFβ/WNT and AKT/MYC pathways also contributing to its downregulation, indicating disrupted fatty acid oxidation in liver cancer (Gopal et al., 2017). ACSM3 may impede the metastasis of HCC cells through the suppression of WNK1-AKT signaling, although it does not influence the proliferation of these cancerous cells (Ruan et al., 2017). Recent studies have identified ACSM3 as a prospective biomarker linked to immune infiltration in liver cancer, making it a viable candidate for early detection and evaluation of immune cell infiltration (Guo X. et al., 2022). In summary, ACSM3 emerges as a novel prognostic indicator for liver cancer and presents itself as a promising therapeutic target.

#### 3.2.3 ACSMs in prostate cancer

Numerous investigations have demonstrated *ACSM1* is significantly upregulated in prostate cancer, with an even more pronounced increase observed in metastatic cases. A comprehensive pan-cancer analysis indicated that ACSM1 exhibited the highest expression levels in prostate cancer, showcasing exceptional sensitivity and specificity as a biomarker (Guo Y. et al., 2022; Shrestha et al., 2024; Alinezhad et al., 2016). Shrestha et al. discovered that androgens can directly bind to the proximal cis-acting element of ACSM1, thereby modulating its expression in prostate cancer (Shrestha et al., 2024). *In vitro* experiments revealed that silencing the ACSM1 gene results in growth cessation, ATP depletion, and the accumulation of polyunsaturated fatty acids within prostate cancer cells. Metabolomic analyses suggest that these cells adapt to the loss of ACSM1 by relying on glycolysis. Furthermore, the *ACSM1* gene appears to bolster the resistance of prostate cancer cells to ferroptosis and facilitate distant cellular metastasis (Shrestha et al., 2024; Alinezhad et al., 2016). Besides, oncogenic influence of ACSM1 in prostate cancer primarily operates through metabolic pathways, while showing no association with immune signaling within the microenvironment. It is also posited that ACSM1 may represent a novel oncogene, serving as a potential biomarker or therapeutic target for the screening and prognostic evaluation of prostate cancer (Guo Y. et al., 2022).

ACSM3 is elevated in prostate tumors, with its expression in prostate cancer surpassing that found in other malignancies. The Androgen receptor (AR) serves as the principal regulator of lipid metabolism within prostate cancer. It directly associates with the proximal cis-regulatory element of ACSM3 to modulate its expression. ACSM3 is integral to the provision of energy through fatty acid oxidation in prostate cancer, and its deficiency may result in the buildup of polyunsaturated fatty acids alongside an upsurge in glycolysis within this cancer type. Besides, the overexpression of ACSM3 has the potential to bolster resistance of prostate cancer cells to ferroptosis (Shrestha et al., 2024).

#### 3.2.4 ACSMs in malignant melanoma

*ACSM3* expression is diminished in malignant melanoma, and its reduced levels are linked to an unfavorable prognosis for the disease (Zhu et al., 2020; Zhao et al., 2022). Zhu et al. established that ACSM3 is correlated with immune rejection in malignant melanoma. ACSM3 overexpression can hinder proliferation, colony formation and invasion of malignant melanoma, while also promoting apoptosis, and it can act synergistically with the BRAF inhibitor PLX-4720 to suppress malignant melanoma growth (Zhu et al., 2020). Research conducted further elucidated the regulatory role of ACSM3 in melanoma. They discovered KLF10 enhances the transcription of ACSM3 in malignant melanoma by binding to its promoter (Zhao et al., 2022). Additionally, KLF10 can elevate ACSM3 levels via PI3K/AKT signaling, thereby impeding the malignant progression of melanoma.

#### 3.2.5 ACSMs in ovarian cancer

The expression of ACSM3 in ovarian cancer tissues is diminished, and individuals exhibiting low levels of ACSM3 demonstrate a less favorable prognosis (Zhang et al., 2021). The overexpression of ACSM3 attenuates the malignant characteristics of ovarian cancer by inhibiting integrin β1/AKT signaling pathway (Yan et al., 2021). ACSM3 influences the metabolic processes of ovarian cancer cells, resulting in an elevated AMP/ATP ratio and the activation of AMPK, thereby reducing the occurrence of high- grade serous ovarian cancer through the enhancement of AMPK activity (Yang et al., 2022). The inactivation of TP53 correlates with resistance to taxane treatment in ovarian cancer cells. The expression profile of TP53, particularly in ACSM3 overexpression, has potential to serve as a predictive biomarker for taxane resistance in ovarian cancer (Shu et al., 2022).

#### 3.2.6 ACSMs in lung adenocarcinoma

ACSM5 is lowly expressed in high-risk patients with lung adenocarcinoma (LUAD) and has been identified as one of the eight genetic characteristics of LUAD. It can accurately provide prognosis prediction for patients with LUAD and is also related to immune infiltration of LUAD (Ma et al., 2020). It indicates that ACSM5 may play a role in the progression of lung cancer.

#### 3.2.7 ACSMs in papillary thyroid carcinoma (PTC)

ACSM5 is downregulated in papillary thyroid carcinoma (PTC), and individuals exhibiting low levels of ACSM5 expression face a bleak prognosis. Consequently, ACSM5 has the potential to act as a prognostic biomarker for patients with PTC. The expression of ACSM5 in PTC patients is influenced by m6A modification, with methyltransferase 3 (METTL3) possibly hindering the advancement of thyroid cancer by enhancing the methylation levels of ACSM5 (Ruan et al., 2021).

Overall, there are not many reports on the involvement of the ACSM family in fatty acid metabolism. Based on the above existing information, there are mainly the following aspects:

1. Activation of medium-chain fatty acids and energy supply:

ACSM1 and ACSM3, as acyl-CoA synthetases, catalyze the conversion of MCFA into acyl- CoA derivatives, driving fatty acid oxidation (FAO) for energy supply. This process is a core pathway of energy metabolism in prostatic cancer cells, and its silencing leads to mitochondrial oxidative stress and disruption of energy supply (Zhao et al., 2022). In LIHC, ACSM3 is also directly associated with the fatty acid oxidation process (Guo Y. et al., 2022).

2. Synergistic regulation with transcription factors:
  2.1. Androgen receptor (AR) regulation: In prostate cancer, AR directly upregulates the expression of ACSM1/3, forming AR-ACSM metabolic axis and promoting tumor growth (Zhao et al., 2022).
  2.2. HNF4α and PPARγ antagonism: In LIHC, HNF4α positively regulates the transcription of ACSM3, while PPARγ inhibits its expression, forming a dynamic balance (Guo Y. et al., 2022).
  2.3. Signaling pathway crossover: The TGFβ, WNT and AKT pathways can downregulate ACSM3, indicating that it is regulated by the integration of multiple signaling networks (Guo Y. et al., 2022).
3. The effect of resisting lipid toxicity stress:

ACSM1/3 protects cells from lipid toxicity caused by the accumulation of medium-chain fatty acids by maintaining the homeostasis of acyl-CoA. Its overexpression can inhibit ferroptosis, and mechanism involves reducing mitochondrial oxidative stress and lipid peroxide accumulation (Zhao et al., 2022).

4. The overall association with the lipid metabolism network:

Silencing ACSM1/3 significantly reshapes the lipidome of prostate cancer cells and affects the abundance of multiple lipid metabolites. For instance, the obstruction of FAO may lead to an enhanced compensatory metabolism of long-chain fatty acids (Zhao et al., 2022).

The summary of the above information is presented in Supplementary Table S2. In addition, based on the above findings and relevant information, we summarized and generalized the distribution of ACSM enzymes and their unique physiological functions, and the related functions of ACSMs, involved preclinical research in Supplementary Tables S3 and S4. Besides, subcellular localization analysis of ACSM family proteins was demonstrated in Supplementary Table S5.

## 4 Future research directions and challenges

### 4.1 Development of small molecule inhibitors

In the treatment targeting the ACSM gene, the development of small molecule inhibitors is an important direction. The existing inhibitor screening mainly focuses on high-throughput screening methods, which can identify and screen compounds with potential activity and evaluate their specific inhibitory effects on ACSMs. For example, developing small molecule inhibitors targeting specific ACSM subtypes can effectively reduce their expression in tumor cells, thereby affecting lipid metabolism reprogramming. During the screening process, researchers also need to pay attention to the biocompatibility and pharmacokinetic properties of candidate compounds to ensure their safety and effectiveness in clinical applications.

According to the existing literature, specific inhibitors targeting the ACSM gene family have not been reported or studied in detail. Most literature discusses the functions and regulatory mechanisms of ACSM enzymes, but does not explicitly address the development or screening of inhibitors (Parsazad et al., 2023). Furthermore, there are significant gaps in the systematic study of the biochemical characteristics of ACSM enzymes (such as substrate specificity and kinetic data), which indirectly indicates a lack of research on inhibitors. Therefore, specialized research on ACSM inhibitors is still in its infancy. Future biochemical and pharmacological experiments may be needed to explore potential inhibitory compounds, especially given the key role of ACSM enzymes in metabolic pathways. Current data suggest that the glycine conjugation process (a two-step detoxification mechanism) involving ACSM genes has not been fully elucidated, which limits the design of potential inhibitors.

With the in-depth understanding of the ACSMs structure, direction of structure optimization has gradually become clear. Through computer-aided drug design technology, researchers can more accurately identify the binding sites with the ACSM domain and design inhibitors with higher affinity. These structure optimizations can be achieved through modifying known small molecule structures and synthesizing new compounds to enhance their inhibitory effects and drug stability.

### 4.2 Basic research demands and clinical transformation

In the exploration of tumor lipid metabolism reprogramming, the necessity for foundational research is particularly paramount. Firstly, elucidating subtype-specific roles is pivotal for comprehending distinctions among various tumor types within lipid metabolism reprogramming process. For example, investigating the diverse subtypes of the ACSM family genes can illuminate their functional variances in specific tumor microenvironments. This subtype-focused inquiry facilitates the formulation of more accurate targeted therapeutic strategies, anticipated to enhance treatment efficacy while minimizing adverse effects. Secondly, importance of three-dimensional structural analysis must not be overlooked. By scrutinizing the three-dimensional architecture of the ACSM family genes, a deeper insight into their molecular interactions and their influence on lipid metabolism can be achieved, bearing significant implications for novel drug development. Furthermore, dynamic regulation visualization technology will afford researchers the ability to observe in real-time the dynamic alterations occurring during the lipid metabolism reprogramming process, thus providing an empirical foundation for further mechanistic investigations.

In the endeavor to transform the research findings concerning the ACSM family genes into tangible clinical applications, the standardization of biomarkers presents a significant challenge. Currently, there is an absence of cohesive standards and protocols for biomarkers across various tumor types, complicating the assurance of consistency and dependability of biomarkers among diverse laboratories and research institutions. Moreover, the development of patient stratification strategies is also confronted with obstacles. Given the inherent variability of tumors, the effective categorization of patients to enable the selection of the most suitable treatment strategy remains a pressing issue in the contemporary research. Future investigations could delve into patient stratification methodologies grounded in the gene expression of the ACSM family to enhance the personalization of clinical therapies.

### 4.3 Research directions of ACSMs in tumors

Most early investigations into the ACSM family predominantly examined their biochemical and enzymatic characteristics, along with their links to non-neoplastic disorders. With exception of ACSM2A and ACSM5, which have yet to have their enzymatic roles elucidated, the remaining members of the ACSM family typically exhibit the function of medium-chain fatty acid:CoA ligase. Notably, ACSM1 and ACSM2B also demonstrate considerable xenobiotic: CoA ligase activity, with benzoate, salicylate, and laurate serving as their principal substrates.

**FIGURE 2:**
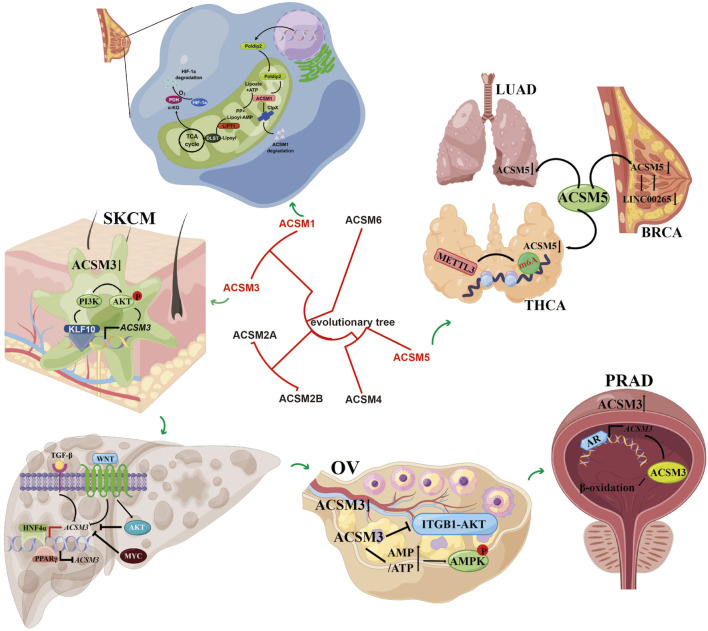
The main functions and involved signaling pathways of the ACSM family genes in various tumors. It demonstrated the mechanism of action of ACSM members in different types of cancer and their related signaling pathways. The figure contains more sections, each representing different types of cancer and related cellular processes. At the center, there is an evolutionary tree that shows the relationships among the members of the ACSM family (ACSM1 to ACSM6). This indicates the evolutionary relevance of these members.

In recent years, a growing array of investigations has concentrated on the involvement of the ACSMs family in the progression of cancer. As mentioned earlier, this article summarizes the roles of ACSM family members in different tumors. ACSM1 expression is notably elevated in both breast cancer and prostate cancer, functioning as an oncogene. ACSM2A and ACSM2B have seen limited research within realm of oncology and are primarily recognized for their heightened expression levels in normal human liver compared to HCC cell lines. Conversely, ACSM3 may assume diametrically opposed roles as either an oncogene or a tumor suppressor, contingent upon the specific type of cancer and its tissue environment. The expression of ACSM4 is significantly linked to unfavorable prognoses in TNBC and, in conjunction with SPDYC, can serve as a genetic marker indicative of poor outcomes in TNBC. ACSM5 is downregulated in LUAD, PTC, and breast cancer, suggesting its potential utility as a biomarker for the prognosis and diagnosis of these conditions. The molecular signals involved in ACSMs in different tumors are shown in Figure 2.

The function of ACSMs in cancer, as indicated through the aforementioned reports, remains inadequately explored. Consequently, we propose that ACSMs harbors potential as diagnostic and prognostic indicators across various malignancies. To investigate this, we assessed the expression levels of the ACSM family members (ACSM1, ACSM2A, ACSM2B, ACSM3, ACSM4, and ACSM5) in tumor versus normal tissues, with data analyzed through the TIMER2.0 database. Findings from this analysis highlighted a marked reduction in the transcriptional levels of ACSMs in a range of cancerous tissues compared to their normal counterparts, with all ACSM members exhibiting downregulation specifically in LUSC (Supplementary Figures S1A, B). These results imply that tumor tissues may diminish the availability of medium-chain fatty acids through a reprogramming of lipid metabolism, warranting further investigation into the underlying causes. Furthermore, a correlation analysis of ACSMs revealed a positive interrelationship among the members (Supplementary Figure S1C).

Further, using the Kaplan-Meier Plotter database, we explored the correlation between the expression of ACSMs demonstrating varied expression in the preceding tumor and the prognostic outcomes in various cancers. The analyses of the KM curve test revealed that ten distinct ACSM- tumor combinations exhibited significant prognostic implications (Supplementary Figure S2). These findings underscore the prospective prognostic importance of ACSMs across a range of malignancies, particularly in KIRC and LIHC.

Numerous investigations have indicated that the reprogramming of lipid metabolism can influence the functionality of immune cells. Consequently, to further substantiate the association between the expression of ACSMs and the infiltration of immune cells in cancer, we employed TIMER to assess the correlations between ACSMs expression, tumor purity, and the levels of immune cell infiltration. Notably, in PAAD and STAD, a significant positive correlation was observed between the expression of ACSM5 and the infiltration of CD8^+^ T cells (r = 0.38, p = 2.58e−07; r = 0.475, p = 2.91e−22), CD4^+^ T cells (r = 0.4, p = 7.23e−08; r = 0.417, p = 7.44e−17), macrophages (r = 0.631, p = 2.19e−20; r = 0.588, p = 9.68e−36), neutrophils (r = 0.473, p = 6.56e−11; r = 0.463, p = 3.98e−21), and dendritic cells (DCs) (r = 0.503, p = 2.27e−12; r = 0.647, p = 2.44e−45), respectively (Supplementary Figure S3). These results imply that ACSM5 may play a crucial role in the immune infiltration of PAAD and STAD.

Summarizing the present discoveries regarding the ACSMs family in cancer research, it becomes evident that this family’s significant involvement in the progression of various malignancies is critical, revealing that genes associated with lipid metabolism also play a role in cancer pathogenesis. Nevertheless, investigations into the ACSMs family in the context of cancer are still insufficient. Certain members remain ambiguous regarding their involvement in cancer. Most of the members that have been definitively linked to cancer have been primarily examined for variations in expression and prognostic significance. To further elucidate their distinct functions across diverse tumors, a more refined analysis is essential, exploring different metabolic pathways, mechanisms of action, or other potential influencing factors to identify the relevant metabolic targets aimed at enhancing tumor diagnosis, treatment, and prognostic outcomes.

## 5 Conclusion

In recent years, tumor metabolic reprogramming has emerged as a pivotal frontier in cancer research, with the ACSM family playing an essential role in this phenomenon. By modulating fatty acid metabolism, the ACSM gene not only influences tumor initiation and progression but is also intricately linked to the malignant characteristics of tumors. While existing studies indicate promising preclinical prospects for therapeutic approaches targeting ACSM, numerous challenges persist in their practical implementation. For example, the functional distinctions among various subtypes of the ACSM gene family remain inadequately defined, complicating the design of effective targeted treatment strategies. Furthermore, there is an urgent need for additional research support regarding treatment strategies aimed at ACSM, specifically in terms of specificity, refinement of delivery systems, and the development of combination therapy regimens. These obstacles not only impede the therapeutic potential of ACSM as a target but also restrict our comprehensive understanding of tumor metabolic reprogramming.

In forthcoming investigations, a thorough examination of distinct functions of each ACSM subtype in tumor metabolism, alongside an elucidation of the regulatory framework governing the ACSM gene in the genesis and progression of tumors, will enable researchers to gain deeper insights into the mechanisms by which tumor cells exploit fatty acid metabolism for their proliferation and survival. Comprehensive studies on this phenomenon will not only facilitate the identification of novel biomarkers but may also catalyze the development of innovative anti-tumor therapeutics.
