# Supplementary material for: Involvement of ACSM family genes in the reprogramming of lipid metabolism within tumors and their investigation as promising therapeutic targets
Source: Front Cell Dev Biol. 2025 Sep 26;13:1663033. doi: 10.3389/fcell.2025.1663033 (PMC12511025; doi:10.3389/fcell.2025.1663033)
Supplement: Supplementary file 1 [file DataSheet1.docx]

**Supplementary Table 1** Properties of human acyl-CoA synthetase medium chains

| **ACSM** | **Alternative Symbols and Aliases** | **Nucleotide reference sequence** | **Protein reference sequence** | **Location** | **Exons** | **Strand** | **Amino Acids** |
| --- | --- | --- | --- | --- | --- | --- | --- |
| ACSM1 | BUCS1; MACS1; LAE; HXM-B | NM_052956.3 | NP_443188.2 | 16p12.2 | 13 | - | 577 |
| ACSM2A | LOC123876; A-923A4.1; MACS2 | NM_001308172.2 | NP_001295101.1 | 16p12.3 | 15 | + | 577 |
| ACSM2B | HXMA; HYST1046 | NM_182617.4 | NP_872423.3 | 16p12.3 | 15 | - | 577 |
| ACSM3 | SAH; SA | NM_005622.4 | NP_005613.2 | 16p13.11 | 14 | + | 586 |
| ACSM4 | LOC341392 | NM_001080454.2 | NP_001073923.1 | 12p13.31 | 13 | + | 580 |
| ACSM5 | FLJ20581; MACS3 | NM_017888.3 | NP_060358.2 | 16p12.3 | 14 | + | 579 |
| ACSM6 | C10orf129, bA310E22.3 | NM_207321.3 | NP_997204.2 | 10q23.33 | 11 | + | 480 |

**Supplementary Table 2** Metabolic functions and regulatory mechanisms of ACSM1 and ACSM3

| Category | Description |
| --- | --- |
| Role in Fatty Acid Metabolism | ACSM1 and ACSM3 function as acyl-CoA synthetases that activate medium-chain fatty acids (MCFAs) to form acyl-CoA derivatives, which are then utilized in fatty acid oxidation (FAO) for energy production. This process supports cancer cell growth and survival by enhancing ATP generation. |
| Regulation of ACSM Expression | In prostate cancer, ACSM1 and ACSM3 are upregulated by the androgen receptor (AR), forming an AR-ACSM axis that promotes metabolic reprogramming. In liver cancer, ACSM3 expression is positively regulated by HNF4α and negatively regulated by PPARγ, and it can be downregulated by signaling pathways such as TGFβ, WNT, AKT, and MYC. |
| Impact on Lipid Metabolism | ACSM1 and ACSM3 are major regulators of the lipidome; their loss leads to metabolic dysregulation, including impaired FAO, mitochondrial oxidative stress, and lipid peroxidation. This indicates their central role in maintaining lipid homeostasis, potentially affecting downstream enzymes involved in β-oxidation or antioxidant defense, though specific enzyme interactions are not detailed. |
| Interactions with Metabolic Pathways | While direct interactions with other lipid metabolic enzymes are not explicitly described, ACSM1/3 activity influences broader metabolic networks. For example, their silencing disrupts FAO, which may indirectly affect enzymes like those in the acyl-CoA synthetase long-chain (ACSL) family or peroxisomal enzymes, but this is inferred from metabolic outcomes rather than proven interactions. |
| Clinical and Therapeutic Relevance | ACSM1 and ACSM3 contribute to therapy resistance (e.g., to antiandrogens in prostate cancer) by supporting energy metabolism and inhibiting ferroptosis, a process linked to lipid peroxidation. In liver cancer, low ACSM3 expression correlates with advanced disease and poor survival, highlighting its role in metabolic perturbations. |

**Supplementary Table 3** Systematic Summary of ACSM Enzyme Distribution and Unique Physiological Functions

| Enzyme | Distribution | Unique Physiological Function |
| --- | --- | --- |
| ACSM1 | Mainly highly expressed in human liver compared to cell lines; upregulated in prostate cancer tissues. | Activates medium-chain fatty acids for mitochondrial oxidation and energy production in the liver; in prostate cancer, it promotes tumor cell growth, lipid metabolism reprogramming, and resistance to ferroptosis via fatty acid oxidation pathways. |
| ACSM2A | Primarily expressed in human liver, with lower levels compared to ACSM2B; methylation changes detected in blood cells in contexts like cardiovascular disease. | Activates medium-chain fatty acids and xenobiotics for detoxification and fatty acid metabolism; genetically conserved with a secondary role in xenobiotic processing compared to ACSM2B. |
| ACSM2B | Highly abundant in human liver mitochondria; dominant transcript in liver tissue. | Primary enzyme for activating xenobiotics and medium-chain fatty acids; essential for glycine conjugation detoxification pathway and maintaining mitochondrial CoA homeostasis; highly conserved sequence suggests a critical role in liver metabolism. |
| ACSM3 | Expressed in liver, but less abundantly than ACSM2B; upregulated in cancers such as prostate cancer and colon adenocarcinoma. | Facilitates medium-chain fatty acid activation for oxidation and energy production; in cancer contexts (e.g., prostate and colon), it supports tumor progression by enhancing lipid metabolism and fatty acid oxidation, and serves as a prognostic biomarker for poor survival outcomes. |
| ACSM4 | Expressed in blood and immune-related cells; polymorphisms studied in peripheral blood mononuclear cells of HIV patients. | Polymorphisms are associated with rapid progression of AIDS in HIV-infected individuals, likely through impacts on immune response or cellular metabolism, distinguishing it from other ACSM enzymes with stronger metabolic roles. |
| ACSM5 | Detected in liver; methylation states altered in blood in cardiovascular studies; differentially expressed in colon adenocarcinoma. | Involved in medium-chain fatty acid activation; in colon cancer, it functions as a diagnostic and prognostic biomarker due to differential expression linked to survival risk, with minor contributions to fatty acid metabolism. |
| ACSM6 | Highly upregulated in bladder cancer (BLCA) tissues compared to normal tissues; enriched in BLCA cells. | Promotes cancer progression by inhibiting CD8+ T cell chemotaxis and anti-tumor immunity, establishing a non-inflammatory tumor microenvironment; contributes to tumor proliferation, migration, and resistance to immunotherapy and other treatments, reflecting its unique role in immune modulation rather than core fatty acid oxidation. |

**Supplementary Table 4** ACSM Enzymes: Tumor Mechanisms and Preclinical Evidence

| Enzyme | Tumor Type | Key Function in Tumors | Preclinical Evidence |
| --- | --- | --- | --- |
| ACSM1 | Prostate Cancer | Activates medium-chain FAs to fuel FAO; Drives metabolic reprogramming; Confers resistance to ferroptosis & anti-androgens | • *In vitro* **knockdown reduces proliferation** in LNCaP cells; Overexpression incr**eases energy production by FAO** and enzalutamide resistance |
| ACSM3 | Prostate Cancer; Colon cancer | Supports tumor growth via FAO; Biomarker for poor prognosis | • *In vivo* shRNA silencing reduces orthotopic xenograft growth; TCGA analysis: High expression correlates with worse survival (HR=1.8, *P*<0.01 in colon cancer) |
| ACSM5 | Colon Adenocarcinoma | Potential diagnostic/prognostic biomarker; Modulates lipid metabolism | • ROC analysis: Diagnostic AUC=0.91 in TCGA cohort; High expression predicts poor response to chemotherapy |
| ACSM6 | Bladder Cancer (BLCA) | Promotes immunosuppressive TME; Inhibits CD8⁺ T-cell activity | • Single-cell sequencing: ↑ACSM6 **linked to ↓CD8⁺ T infiltration; CRISPR knockout in BLCA models** anti-PD-1 efficacy |

**Supplementary Table 5** The localization of ACSMs within cells

| **Gene Name** | **Primary Localization / Functional Correlation** | **Reference** |
| --- | --- | --- |
| ACSM1 | **Mitochondria and Cytoplasm** (exhibits subcellular localization shifts); Functionally linked to fatty acid metabolism and mitochondrial β-oxidation**)** | **[**12**]**, **[**14**]** |
| ACSM2A | Not mentioned | - |
| ACSM2B | **Mitochondria (**ACSM2B, initial isolated from human liver mitochondria) | **[**18**], [**19**]** |
| ACSM3 | **Closely associated with mitochondrial function** (its deficiency causes mitochondrial dysfunction); Functionally involved in mitochondrial fatty acid metabolism | **[**21**]** |
| ACSM4 | **Mitochondria** (ACSM4 belongs to the mitochondrial proteins encoded in the cell nucleus (NEMPs) | **[**30**]** |
| ACSM5 | Function highly correlated with mitochondrial metabolism (regulates fatty acid oxidation, induces ferroptosis); Specific subcellular localization not explicitly described | **[**37**]** |
| ACSM6 | Not mentioned | - |

**Supplementary Figure 1**


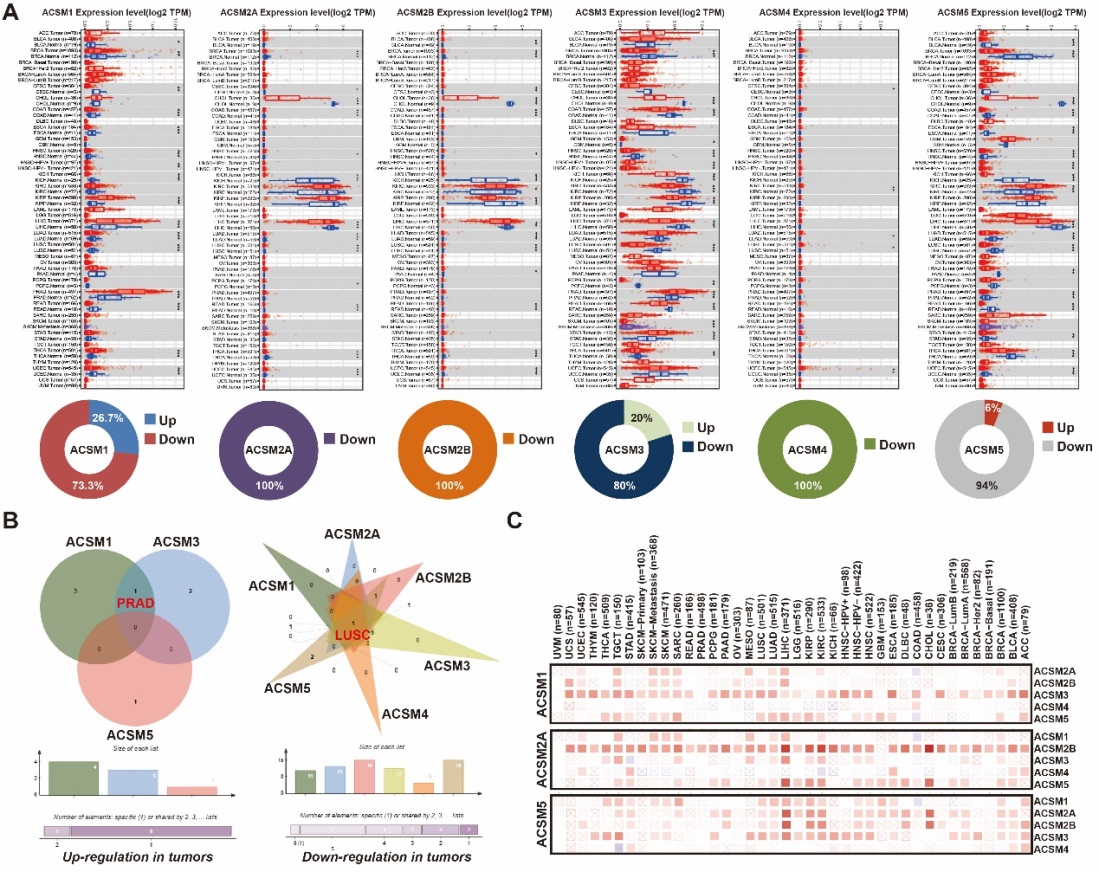


**Supplementary Figure 1**. The expression levels of ACSM family in various types of tumor and normal tissues. (**A**) Expression levels of ACSMs in various normal or cancer tissues by TIMER2.0 analysis. (**B)** Venn diagram of the cancers showing differential expression of ACSMs selected by TIMER2.0. **(C)** Correlation analysis among ACSM members.

**Supplementary Figure 2**

**
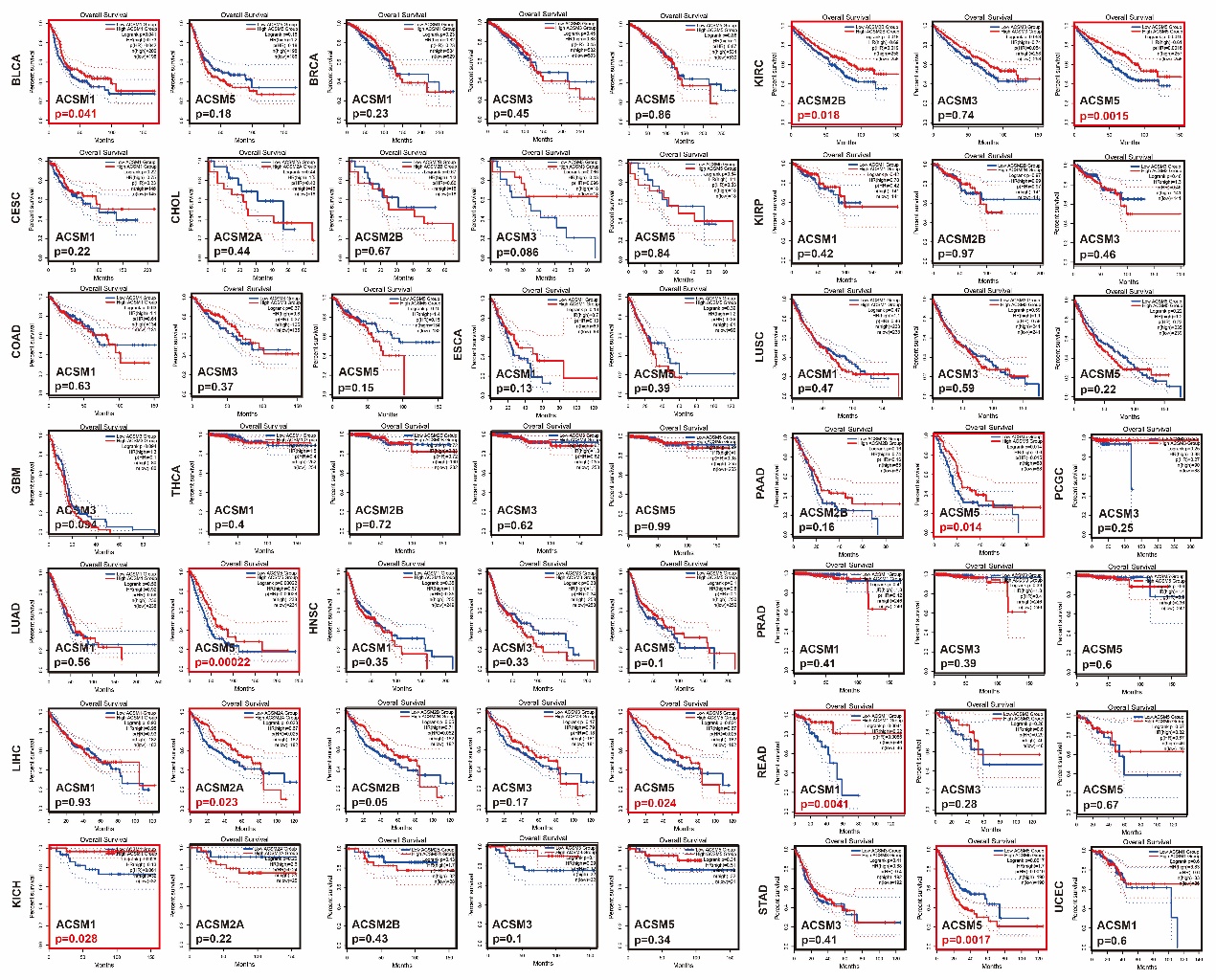
**

**Supplementary Figure 2**. Correlation analysis between the ACSMs expression and prognostic survival in various cancer patients through Kaplan–Meier plotter analysis (the red flags indicate prognostic differences).

**Supplementary Figure 3**

**
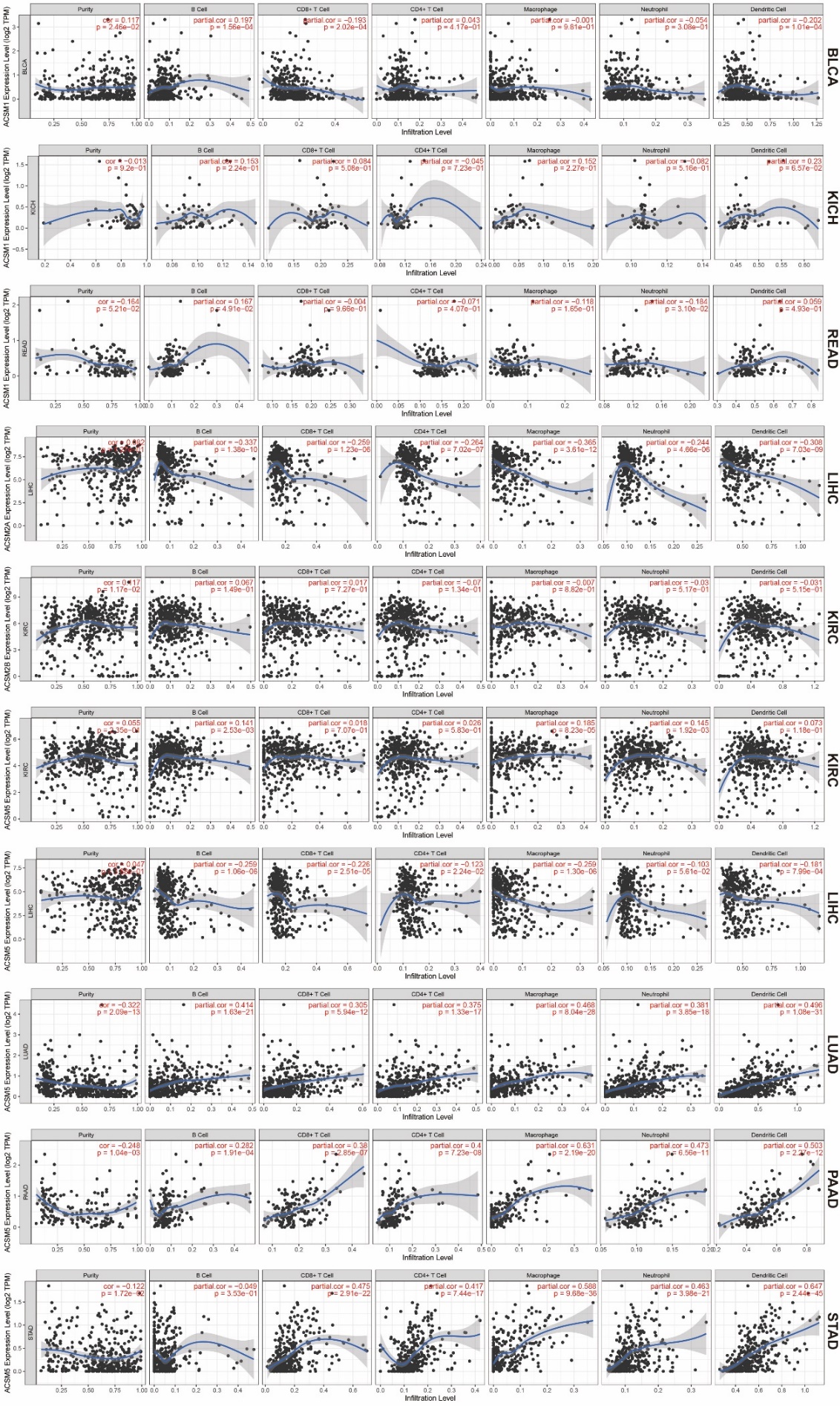
**

**Supplementary Figure 3**. Correlation analysis between ACSMs expression and immune cell infiltration levels in various cancer tissues analyzed via TIMER.
